# Acquired Tracheoesophageal Fistulas: A Case Report and Review of Diagnostic and Management Challenges

**DOI:** 10.7759/cureus.23324

**Published:** 2022-03-19

**Authors:** Leen Hasan, Bashar Sharma, Steven A Goldenberg

**Affiliations:** 1 Internal Medicine, University of Connecticut Health, Farmington, USA; 2 Gastroenterology and Hepatology, University of Connecticut Health, Farmington, USA

**Keywords:** dual stenting, tracheal stenting, esophageal stenting, tracheostomy, tracheoesophageal fistula

## Abstract

Acquired, nonmalignant tracheoesophageal fistulas (TEFs) often occur in the setting of prolonged use of endotracheal or tracheostomy tubes due to trauma and erosion of the tracheal wall inflicted by tube cuffs or direct tracheal contact. In this report, we present a patient with a tracheostomy who presented with recurrent aspiration pneumonia and was found to have a large TEF that was difficult to treat. We also discuss the diagnostic and management challenges concerning TEFs. TEFs, especially if large, lead to recurrent aspiration pneumonia and can be challenging to manage. Definitive management of TEFs involves surgical repair; meanwhile, endoscopic or bronchoscopic stenting to bypass the fistula can be performed. The fistula location, size, and concurrent positive pressure ventilation make its treatment challenging in those with chronic ventilatory dependence. Early recognition and multidisciplinary management involving gastroenterologists, interventional pulmonologists, and thoracic surgeons are necessary to decide on the best treatment strategy.

## Introduction

Tracheoesophageal fistula (TEF) is an abnormal opening that forms between the trachea and esophagus and occurs most commonly as a congenital phenomenon that manifests soon after birth with respiratory and gastrointestinal (GI) complications. In adults, most TEFs are acquired and often occur in the setting of esophageal or lung cancer, which accounts for more than half of TEFs [1]. Acquired, nonmalignant TEFs are due to prolonged endotracheal intubations, trauma, caustic ingestions, endoscopic or surgical interventions, radiotherapy or infectious, and inflammatory diseases [1]. Because they are rarely encountered in adults, TEFs are often difficult to diagnose and can present with recurrent cough, pneumonia, and unexplained malnutrition. When prolonged endotracheal intubation is the cause (i.e., prolonged tracheostomy), patients present with worsening of oxygenation due to loss of tidal volume ventilation and recurrent aspirations. Endoscopic and surgical measures can be undertaken as viable treatment options to prevent life-threatening trachea-bronchial contamination and malnutrition. Several diagnostic and treatment challenges exist, especially among patients who are on chronic ventilatory support since positive pressure ventilation after surgical repair results in anastomotic dehiscence. In this article, we report a case of a large TEF in a patient with chronic tracheostomy that was refractory to treatment. Then, we discuss the diagnostic and treatment challenges of TEFs, including updates in the curative and palliative approaches to treating this population of patients.

This article was previously presented as a meeting abstract at the 2021 American College of Gastroenterology Annual Scientific Meeting on October 24, 2021.

## Case presentation

**Figure 1:**
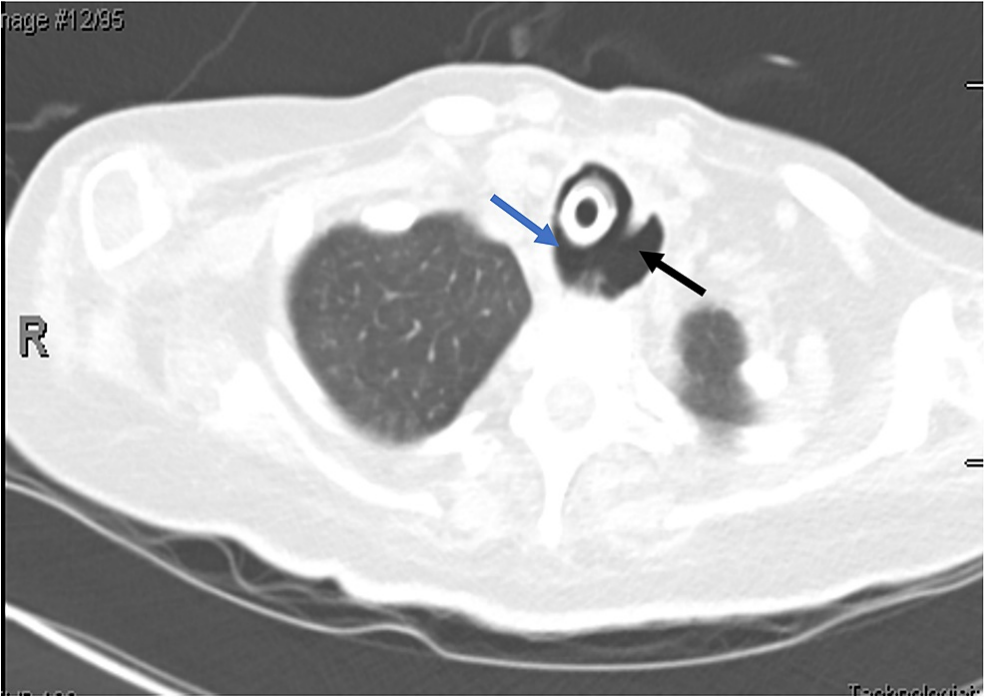
Tracheoesophageal fistula on chest CT. CT scan of the chest reveals tracheoesophageal fistula (blue arrow: trachea, black arrow: esophagus). CT: computed tomography.

A 26-year-old female with a history of anterior spinal artery embolism resulting in quadriplegia and chronic ventilatory support (status post-tracheostomy and gastrostomy tube placement) presented with confusion and increased secretions from the tracheostomy site. She was found to be hypercapnic, which was thought to be due to inadequate delivery of tidal volumes through the tracheostomy and the cause of the change in her mental status. Chest computed tomography (CT) scan revealed an abnormal dilation of the lower esophagus and upper thoracic trachea along with fistula formation (Figure 1), as well as diffuse patchy ground-glass opacities representing aspiration pneumonia, for which she was started on broad-spectrum antibiotics and admitted to the intensive care unit (ICU) for ventilatory support. 

**Figure 2:**
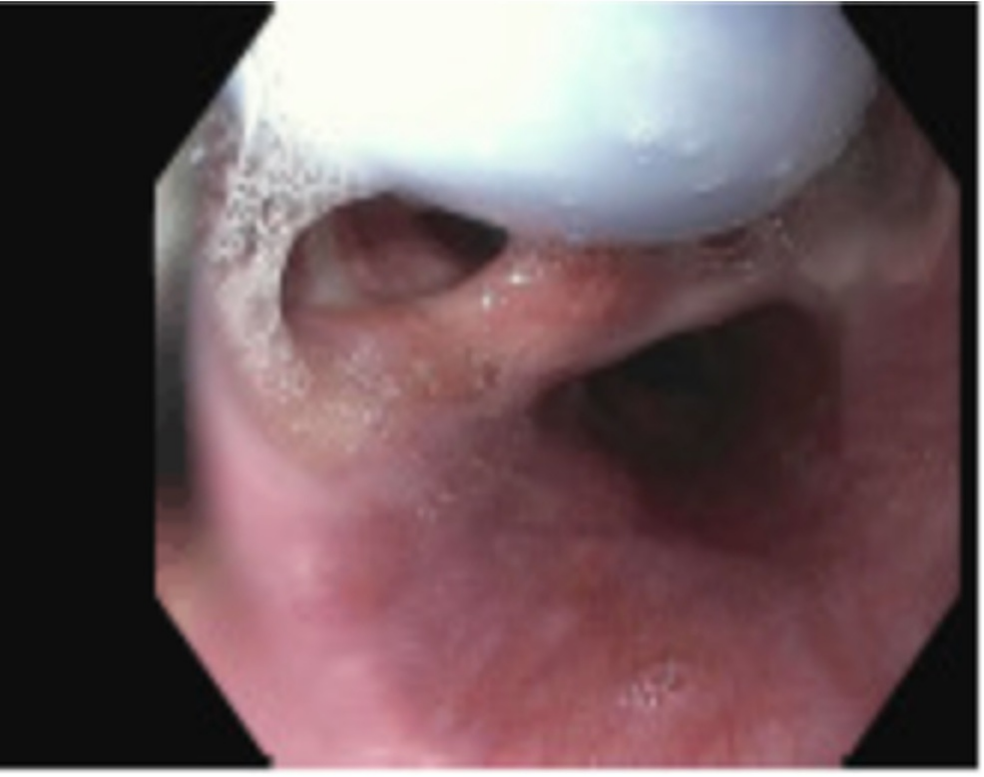
Tracheoesophageal fistula on upper endoscopy. Upper endoscopy demonstrating a tracheostomy balloon in the upper esophagus 20 mm from the incisors just below the upper esophageal sphincter. There is a widely patent tracheoesophageal fistula large enough to allow passage of the tracheostomy balloon.

Given the findings on chest CT, a bedside bronchoscopy was performed and showed a tracheal diverticulum with a fistulous tract into the esophagus, consistent with TEF, which was later further confirmed on an esophago-gastro-duodenoscopy (EGD) (Figure 2). On EGD, the fistula was found to be too large to be amenable to endoscopic stenting. Throughout her stay, the patient’s ventilation was position-dependent to maintain adequate tidal volumes, requiring several bedside bronchoscopies to adjust and advance the tracheostomy tube, with only temporary improvement. Eventually, the interventional pulmonology team deployed a Y silicone stent into the airway under bronchoscopy guidance to bypass the fistula and promote healing, followed by placing a tracheostomy tube through the Y stent stoma site. This successfully improved tidal volume delivery, and the patient was discharged to a specialized resident facility after a week of hospital stay. 

Unfortunately, the patient presented again to the emergency room three days after discharge with fever and recurrent aspiration pneumonia, requiring re-admission to the ICU. A repeat bedside bronchoscopy revealed a patent endotracheal stent; however, there were large amounts of tenacious secretions in the bilateral lower lobes, along with inflamed mucosal lining suggestive of pneumonia, likely due to continuous fluid aspiration from the GI tract. She was found to have intermittent air leaks, likely due to inadequate healing of the TEF, which was temporarily corrected by placing the neck in a flexed position. Her hospital course was complicated by recurrent aspiration events and she continued to have intermittent worsening of her ventilation throughout due to aspiration of GI content around the stent into the airway. 

Thoracic surgery was consulted for consideration of esophagectomy to prevent reflux and recurrent aspirations. She was not deemed a candidate for esophagectomy due to chronic ventilation status, in which continuous positive pressure ventilation would result in a permanent air leak if esophagectomy was performed and prevent healing. She was re-evaluated again by interventional pulmonology, along with multidisciplinary, nationwide consultations involving thoracic surgery, pulmonology, and gastroenterology to explore other surgical and endoscopic options, including the placement of a metal stent (to promote more granulation tissue formation around the fistula), and the placement of a wider diameter Y stent to prevent aspirations from around the stent. No open surgical interventions were thought to be helpful, and the patient eventually underwent replacement of the Y silicone stent with a larger diameter stent one month after hospitalization, which improved ventilation, and she was discharged from the hospital. Unfortunately, the patient was re-admitted to the ICU twice in the following weeks due to respiratory distress and recurrent aspiration pneumonia. During her last admission, she was found to have increased airway pressure due to stent migration, and multidisciplinary meetings were held with the family and the patient to explain the limited therapeutic options and the unfortunate discomfort the patient has suffered. The patient’s code status was eventually made comfort-measures only, and she passed away.

## Discussion

In this article, we present a case of a young patient with acquired large TEF due to a chronic tracheostomy tube that was refractory to endoscopic stenting and not amenable to surgical management, who suffered from recurrent aspirations and an inability to achieve adequate ventilatory needs. This case highlights the challenges in the diagnosis and management of this disease entity and the importance of multidisciplinary management and palliative approaches in the care of this patient population. 

Clinical presentation and diagnosis 

It is estimated that tracheal injuries and erosions occur at an incidence of 0.3-3% of mechanically ventilated, secondary to traumatic intubations, aggressive airway suctioning, and tracheal wall ischemia related to local vascular compression by the endotracheal tube (ETT) or the tracheostomy tube or cuff [2]. Symptomatic presentation of TEF usually occurs three to four weeks after a tracheal-cuff-related injury, which hinders prompt diagnosis and management in the early stages [2]. The clinical presentation of TEF varies depending on the location, size, and rate of its formation. The most common presenting symptoms are cough, followed by aspiration, fever, swallowing difficulty, and pneumonia [3]. Uncontrolled coughing after swallowing, known as “Ono’s sign,” is a specific sign of TEF that occurs in nonventilated patients [2]. Early diagnosis of TEF and having a low threshold of suspicion are important in preventing further lung and tracheal injury. Chest imaging, including chest X-ray and CT, often identifies evidence of repeated aspiration, and CT can identify the presence of a tracheal defect. A barium swallow test in patients who are able to take per os (PO) can identify the TEF in 70% of cases [4]. This is usually followed by an EGD to confirm the location and size of the fistula. However, small TEFs can be missed in esophageal folds, which could be easily identified via bronchoscopic evaluation.

Management

Once TEF is diagnosed, efforts are undertaken to minimize aspiration of gastric contents into the airway while evaluating for definitive surgical or endoscopic procedures. This can be achieved by advancing the ETT or tracheostomy tube so the cuff lies below the level of the fistula. However, this is only a temporary measure since aspiration of gastric contents can still occur around the tube and into the airway. Thus, aspiration of gastric content and acid reflux therapy can provide additional temporary protection while awaiting definitive therapy in cases with recurrent aspirations is evident. 

The definitive management of TEFs varies as there is limited data and consensus regarding the best approach. The treatment depends on the TEF etiology, size, and location. In general, management of malignant and benign TEFs not amenable to surgical options focuses on improving the quality of life with palliative measures [5]. Surgical options include primary repair of an esophageal defect, esophageal repair with muscle flap, tracheal resection and anastomosis, tracheal defect repair with muscle flap, and others [6]. Surgical management usually provides definitive therapy. However, morbidity is relatively high, especially in the early post-operative period. One of the barriers to definitive operative management is the concurrent need for chronic mechanical ventilation, in which positive pressure ventilation causes anatomic dehiscence of surgical repair and prevents tissue healing. For this reason, early weaning from positive pressure ventilation and, potentially, extubation should be attempted as soon as possible after surgical management.

Non-operative options include esophageal stents, airway stents, esophageal and airway (i.e., dual) stents, defect suturing and glue injections, and others [5]. Stenting is usually pursued as either (1) a palliative measure in non-operative cases, especially in malignant TEFs, to reduce aspiration, dysphagia and improve nutritional status; (2) a bridge to definitive surgical management [7]. TEFs < 5 mm can be managed with local therapy such as glue injection or suturing, while TEFs > 5 mm are managed with airway and/or esophageal stenting. Single esophageal stenting is pursued in patients with mid-to-distal esophageal defects, utilizing self-expanding metallic stents given their established durability and established efficacy for other esophageal diseases [7]. It is important to note that single esophageal stents may result in external compression of the airway, making it an undesirable option for those with tracheal stenosis or at high risk for airway obstruction. Single esophageal stenting is technically difficult in those with proximal esophageal defects. On the other hand, single airway stents are preferred in patients with pre-existing airway stenosis or those with defects in the proximal trachea. When an airway stent is placed, efforts should be made so that the fistula is fully covered with a covered safety margin of 20 mm at both ends, to prevent further expansion of the fistula secondary to the stent. Dual airway and esophageal stenting are often pursued for mid to distal esophageal TEFs, especially in malignant TEFs, among patients who are at risk of airway compromise. Although some studies showed improved survival in dual versus single stenting approaches, this evidence remains anecdotal [8].

## Conclusions

The involvement of multidisciplinary teams and the consideration of palliative management remain important in the management of TEFs. As in our patient, certain cases of TEFs are very difficult to manage, and establishing goals of treatment with the patients, their families, and other team members early is key. Preventing aspirations and further lung injury while awaiting definitive surgical management is important and can be achieved by esophageal and/or airway stenting in the majority of cases. When these measures are insufficient, high-risk measures such as esophagectomy or tracheal resections are considered, but are not usually viable options in patients with chronic ventilatory dependence. More prospective research and innovative biomedical engineering is required to improve the treatment options of this difficult disease.
